# Disparities in patient-centered communication for Black and Latino men in the U.S.: Cross-sectional results from the 2010 health and retirement study

**DOI:** 10.1371/journal.pone.0238356

**Published:** 2020-09-29

**Authors:** Jamie A. Mitchell, Ramona Perry

**Affiliations:** School of Social Work, University of Michigan, Ann Arbor, MI, United States of America; Universidad del Desarrollo, CHILE

## Abstract

**Background:**

A lack of patient-centered communication (PCC) with health providers plays an important role in perpetuating disparities in health care outcomes and experiences for minority men. This study aimed to identify factors associated with any racial differences in the experience of PCC among Black and Latino men in a nationally representative sample.

**Methods:**

We employed a cross-sectional analysis of four indicators of PCC representative of interactions with doctors and nurses from (N = 3082) non-Latino White, Latino, and Black males from the 2010 Health and Retirement Study (HRS) Core and the linked HRS Health Care Mail in Survey (HCMS). Men’s mean age was 66.76 years. The primary independent variable was Race/Ethnicity (i.e. Black and Hispanic/Latino compared to white males) and covariates included age, education, marital status, insurance status, place of care, and self-rated health.

**Results:**

Bivariate manova analyses revealed racial differences across each of the four facets of PCC experience such that non-Hispanic white men reported PC experiences most frequently followed by black then Hispanic/Latino men. Multivariate linear regressions predictive of PCC by race/ethnicity revealed that for Black men, fewer PCC experiences were predicted by discriminatory experiences, reporting fewer chronic conditions and a lack of insurance coverage. For Hispanic/Latino men, access to a provider proved key where not having a place of usual care solely predicted lower PCC frequency.

**Implications:**

Researchers and health practitioners should continue to explore the impact of inadequate health care coverage, time-limited medical visits and implicit racial bias on medical encounters for underrepresented patients, and to advocate for accessible, inclusive and responsive communication between minority male patients and their health providers.

## Introduction and background

Black men and several racially, culturally and ethnically heterogeneous subgroups of Latino men are burdened by pervasive and unrelenting poor health outcomes across socioeconomic levels [1–3]. For example, Mexican, Puerto Rican and other U.S. born Latino men experience significantly higher levels of functional limitations and related disabilities, overweight and obesity, and lower utilization of primary care compared to White males [1, 4]. Nationally representative data reveals that among all Latino men in the U.S., Puerto Rican American men have the highest rates of cardiovascular disease, substance use, depression, smoking, asthma, and arthritis [1].

Health disparities impacting Black men include higher incidence and mortality rates for cardiovascular disease, hypertension, stroke, and several forms of cancer, leading to an estimated $24.2 billion in excess medical expenditures for Black men between 2006 and 2009 alone [5, 6]. Relative to other racial and ethnic groups of men in the U.S., both Black and Latino men also have disproportionately high prevalence rates for diabetes [7, 8], delayed diagnosis, suboptimal treatment outcomes and shorter survival across several types of cancer [9], and often do not meet current recommendations for physical activity which is an important protective factor against chronic disease risk and poor outcomes [10].

These racial/ethnic disparities in minority men’s health and longevity have often been attributed to poor lifestyle choices or health behaviors. For example, behavioral factors such as smoking, alcohol use, physical inactivity, obesity, unhealthy eating, and suboptimal utilization of preventive health care services contribute to poor health outcomes [11, 12]. Minority men’s general disinclination to engage with the health care system has also been linked to medical mistrust resulting from perceived bias by health care professionals, that compounds and reinforces stress on account of racism encountered and internalized in every day interactions [11, 13–17]. Research also demonstrates that the simultaneous experience of multiple levels of racism, such as interpersonal racism and internalizing negative racial bias, can accelerate markers of premature biological aging and contribute to chronic disease and early mortality in Black men [18]. Further, cultural constructions of masculine identity that reinforce harmful aspects of gender norms are shown to negatively impact health help-seeking and utilization of preventive health services for Black and Latino men [16, 17, 19, 20].

There is little dispute that health disparities impacting Black and Latino men are patterned according to well-established social determinants of health such as lower socioeconomic status, higher unemployment, residing in economically disadvantaged areas, mass incarceration, and health policies; leaving minority men especially vulnerable to negative health trajectories [3]. For example, socioeconomic factors such as financial hardship and a lack of health insurance contribute to disparities in healthcare access and utilization among Black and Latino men [21, 22]. Our own research and that of others also finds that patient-provider communication is an underexplored pathway that influences how minority men may experience social determinants of health. Specifically, patient-provider communication has the potential to both directly and indirectly connect patients to health care access, advocacy, resources, self-efficacy and self-management, improved knowledge, shared understanding and health decision-making [23, 24].

In the current study, we are concerned primarily with factors established across the literature base that are shown to shape the experience of patient-centered communication during medical interactions with adult minority male populations. As we will discuss in subsequent sections, many of these factors are individual or psychosocial in nature such as race/ethnicity [25], age and gender [26], or one’s experience with chronic disease [27, 28]; while others are inextricably linked to more systemic determinants of health such as health care access [29] and discrimination [30]. Interestingly, the few existing prior studies that speak to minority men’s experiences with health communication have utilized small and non-representative samples that make it difficult to generalize findings regarding the impact of patient-centered communication on the health and wellbeing of minority men. Here, we attempt to contribute meaningful and actionable context from a nationally representative sample regarding the factors that shape some minority men’s communication experiences with health providers; a critical next step in advancing the knowledge base. Taking into account that Black and Latino men are not a monolith, it is also significant that the current study tackles both within and between group differences that potentially shape Black and Latino men’s vulnerability to suboptimal communication in a health care setting. Again, patient-provider communication is a key pathway through which some health disparities that disproportionately impact Black and Latino men are perpetuated or ameliorated [23]; further necessitating the contribution of this work.

### Patient-centered communication

Patient-centered communication, hereafter referred to as PCC, provides a useful conceptual lens to help inform the current study, which is specifically examining factors associated with indicators of PCC as experienced by between Black and Latino men in nationally representative study. Patient-centered communication refers to a philosophy, practice, and central component of high-quality health care interactions, innovations and services; the term PCC emerged from a report by the Committee on Quality Healthcare in America within the Institute of Medicine in 2001 advising that medical care should become more ‘patient-centered’. Conceptually, PCC includes four overlapping core components; (1) regarding the patient’s unique perspective, needs and preferences, (2) encouraging patients to share power and responsibility for decision-making with their providers as they are capable and willing, (3) promoting shared understanding between patients and providers about the medical issues and treatment, and (4) situating the patient within their individual psychosocial context, including meeting patients’ informational and emotional needs [31, 32]. The National Cancer Institute and other scholars have published a framework and recommendations that better elucidate the domains and sub-domains of PCC, including guidance on how to operationalize complex PCC constructs and functions [31, 33, 34] There is also an extensive body of literature linking physicians’ use of patient-centered communication approaches to patient outcomes subsequent to the medical encounter. For example, the use of active listening and providing detailed and jargon-free information by physicians has been associated with increased patient satisfaction, a key healthcare indicator [35]; while showing empathy and responding to patients’ emotional concerns are PCC behaviors demonstrated to increase patient satisfaction [36]. Several comprehensive reviews have compiled available evidence on the relationship between PCC behaviors by health providers and a range of health outcomes in adult patients, including its suggestive impact on blood pressure, blood glucose, depression, and patient adherence to medical recommendations [32].

When Black and Latino patients interact with health providers, there is evidence, generally, that physicians exhibit less patient-centered communication with them compared to White patients, such as using a harsher tone or providing less time for patients to ask questions; often as a result of implicit racial bias [37–39].

The limited extant research on experiences of PCC for Black men demonstrates that Black men without a spouse or partner, who are older, and those with mental health issues such as depression, are more likely to report barriers to communicating with primary care physicians [24, 26, 40, 41]. Relatedly, a study of unmet health communication needs among Black men concluded that Black men with less education, lower incomes, more comorbidity, and less access to a usual source of health care (i.e. a relationship with a primary care provider for routine, preventive, and chronic disease care) were most likely to report difficulty getting their questions answered by primary care physicians during medical visits; a proxy for patient-centered communication [24]. Considerably less attention has been paid to the disparate PCC experiences for Latino males, and research on Black males is still nascent. This is a significant stumbling block to developing and testing interventions to optimize patient-centered communication, including increased active participation in medical visits, particularly for vulnerable subgroups within these populations.

## Methods

### Dataset and study participants

The present study is a cross-sectional analysis with data drawn from the publicly available 2010 wave of the biennial Health and Retirement Study (HRS) Core Survey, the linked HRS Health Care Mail in Survey (HCMS), and the 2010 HRS Mail in Psychosocial Leave-Behind Survey. All data was available for public use and is supported by the National Institute on Aging and the Social Security Administration. The HRS is a nationally representative panel study of American adults. The study includes five birth cohorts representing Americans born in 1953 and earlier. The HRS assesses important aspects of health and the aging process and has response rates of over 90% across waves. Technical details on the sampling design, recruitment, health measures, and linkage with administrative data for the Health and Retirement Survey have been published extensively and can be found elsewhere [42–46]. The primary core survey was administered to 37,498 respondents. Of these, 29,850 respondents did not have data available in the HCMS file, which contained patient-centered communication measures, and thus were excluded. Of the remaining 7,648 respondents, those who were less than 40 years old (275) and did not identify as male (4,289) were excluded, resulting in a final analytic sample of 3,084 men across all data sets in 2010. As previously noted, minority men in the U.S., and Black men specifically, are subjected to unique socio-cultural experiences such as racism, that may trigger premature aging and mortality, and increased chronic disease [18]. In this study, establishing a slightly younger age criteria for inclusion (i.e. 40 years old) could capture more minority men who experience some of the sequalae associated with premature aging and any related PCC effects, much earlier than the while male reference group in the sample.

### Measures

The primary outcome measure is patient centered communication (PCC) experiences as measured by four items combined into a single continuous scale; a method used across PCC research [47]. Respondents were asked to recall their experiences getting medical care in the previous twelve months. The questions were as follows: (1) How often did the doctors and nurses explain things in a way that is easy to understand? (2) How often did the doctors and nurses listen carefully to you? (3) How often did the doctors and nurses show respect for what you had to say? (4) How often did the doctors and nurses spend enough time with you? Respondents answered using a 4-item response scale of (1) Never, (2) Sometimes, (3) Usually, and (4) Always. The PCC measures are used individually for bivariate analyses and for multivariate analyses, these four questions were combined into a single continuous scale of patient centered communication; a composite measure of PCC is commonly used in similar research [47]. These items have also been used reliably in other research on minority males and PCC [24, 48].

In preparing the PCC continuous scale, principal components analysis was used to identify and compute a composite score for the factors underlying the above items. Initial eigen values supported a single factor solution and indicated that the first factor explained 81% of the total variance. Factor loadings indicated that each of the four items were equally relevant to the factor’s dimensionality. Having confirmed that these four items measure the same underlying construct, Cronbach’s alpha was used to measure the reliability of the scale. The four-item PCC scale was found to be highly reliable (a = 0.92). The four PCC items were then averaged across respondents. This continuous unstandardized mean scale of PCC experiences, ranging from 1–4, is employed as the outcome variable in multivariate analyses. Increasing values on this scale are indicative of more frequent experiences with PCC across the each of the four measures combined.

With regard to primary independent variables, HRS measures of race (Black/African American, White/Caucasian, and other) and ethnicity (Hispanic/Latino, not Hispanic/Latino) were combined to create a race/ethnicity measure with the following categories: non-Hispanic White (N = 2275), non-Hispanic Black (N = 443), and Hispanic/Latino (N = 364). Respondents who racially identified as “other” were removed from the analysis so as not to obscure the results. In multivariate analyses, non-Hispanic White was used as a reference category, a strategy that is consistent with existing research.

Sociodemographic covariates included age in years, marital status, and level of education. Age was measured continuously while marital status included the following categories: married/partnered, divorced/separated/never married, and widowed. Similarly, education was measured categorically by highest level completed: less than high school (less than 12 years of education), high school equivalent (12 years of education), some college (greater than 12 but less than 16 years of education), and college or more (more than 16 years of education).

Our sole psychosocial covariate, perceived discrimination, was captured using the Everyday Discrimination Scale (EDS). Again, discrimination was chosen as a covariate because it is referenced extensively in the literature on patient-centered communication [30, 37–39, 49]. The EDS captures chronic or routine experiences with discrimination (as opposed to major discriminatory experiences) [50]. The EDS is a validated scale that has demonstrated reliability among older adult samples. The EDS is comprised of six questions that probe the frequency of respondents’ experiences with discrimination. Respondents are asked: In your day-to-day life, how often have any of the following things happened to you? (i) “You are treated with less courtesy or respect than other people”; (ii) “You receive poorer service than other people at restaurants or stores”; (iii) “People act as if they think you are not smart”; (iv) “People act as if they are afraid of you”; and (v) “You are threatened or harassed”; (vi) “You receive poorer service or treatment than other people from doctors or hospitals.” Response categories were never, less than once a year, a few times a year, a few times a month, at least once a week, or almost every day.

A perceived everyday discrimination scale was created for the purposes of analysis by coding each item such that increasing values indicate more frequent experiences with discrimination. The items were then averaged to create a mean score for perceived discrimination.

We examined three available biophysical measures in the data set as covariates as patient physical and mental health status have been shown in the literature to be germane to patient-provider communication dynamics [24]. To determine physical functioning we included an assessment of the ability to perform *Activities of Daily Living* (ADLs). Participants were asked if they had difficulty with any of five activities: bathing, dressing, eating, walking, and getting out of bed [51, 52]. Affirmative responses were combined to create a continuous measure ranging from 0–5. As physical health is often viewed as multiaxial [53] we included both subjective and objective assessments of general health [54]. The self-rated overall health status measure asked respondents to characterize their overall health as poor, fair, good, or excellent. For analytic purposes, fair/poor (0) and good/excellent (1) were collapsed to create a dichotomous measure. We also included a measure of the presence of common chronic medical conditions including hypertension, diabetes, cancer, chronic lung disease, heart conditions, stroke, psychiatric problems, and arthritis. Respondents indicated whether a physician had ever told them that they had each condition. Our measure of comorbidities ranged from 0–8 and was a count of affirmative responses to having any of the conditions. The ADL functioning measure and comorbid conditions measure were both coded such that higher values indicated more physical health issues. Finally, we also included a measure of Body Mass Index (BMI) as a proxy for weight status. This continuous measure of BMI was created by HRS by dividing weight (in kilograms) by squared height (in meters).

Depression was the sole psychological covariate. Depression which was measured by affirmative (yes) responses to symptomatic items on the 8-item Center for Epidemiological Studies Depression (CES-D) Scale [55]. Respondents indicated if they had any of the following feelings over the past week prior to their interview: felt depressed, felt that everything was an effort, felt they could not get going, had restless sleep, felt lonely, felt sad, felt happy, and enjoyed life. A continuous summary measure of these items was prepared ranging from 0–8 with increasing numbers indicative of more depressive symptoms. Two items (felt happy and enjoyed life) were reverse coded in accordance with the direction of the final scale measurement.

Two measures were used to measure access to healthcare, a key factor in shaping health care experiences for minority male populations [56]. Place of care was measured by a categorical measure indicating the typical place the respondent sought medical care. Response options include a doctor’s office or HMO, medical clinic, ER, outpatient facility, some other unspecified place, or having no place to go for medical care. We used two measures of insurance for the current study. One is a yes/no measure of whether the respondent has any insurance coverage. We also employed a measure of primary insurance type which included the following response categories: Medicare, Medicaid, employer provided, private insurance, Tri-Care/Champus, some other public source, and Veterans Affairs-provided insurance (VA).

### Statistical analyses

Sample characteristics were summarized using univariate frequency and descriptive statistical procedures. The relationship between race/ethnicity and our four PCC outcomes was initially examined using MANOVA. Specifically, a one-way multivariate analysis of variance (MANOVA) was conducted to test the hypothesis that there would be one or more mean differences between Race/Ethnic groups (non-Hispanic White, Non-Hispanic Black, Hispanic/Latino) on reported PCC experiences. After establishing that statistically significant differences exist by Race/Ethnicity in PCC outcomes, post-hoc comparison testing was conducted to reveal specific group differences in PCC experience. Next, once race/ethnic differences in PCC were confirmed, we then examined the correlates of PCC experiences for men using Ordinary Least Squares (OLS) regression analysis. A series of nested multivariate linear regression models was conducted to examine whether race can significantly predict scores on the PCC experience averaged scale ranging from 1/low frequency of PCC to 4/high frequency of PCC controlling for our covariates. We also sought to identify the unique predictors of PCC experiences for each race/ethnic group and socio-demographic, psychological, health-related, and health access factors that influence men's experiences with PCC. All models employed the same biopsychosocial control measures. Finally, we conducted analyses to identify correlates of PCC experience for Black and Hispanic/Latino respondents for whom analyses have evidenced decrements in PCC experience relative to White respondents. For these investigations, the scaled PCC outcome was again employed. Two separate OLS regression models predictive of PCC experiences were conducted, one focused on Black respondents and the other Hispanic/Latino respondents.

## Results

### Sample characteristics

Characteristics of the study participants are presented in Table 1. Most of the study sample was non-Hispanic White (73.8%) followed by non-Hispanic Black respondents, who comprised just under 15% of the sample. Hispanic/Latino respondents comprised 11% of the sample. Respondents reported an average of 3.41 on the 4-point PCC scale. Respondents were relatively well educated with 49.93% having completed some college or more.

**Table 1:** Sample characteristics for full sample and by race/ethnicity. HRS, HCMS, PLBS 2010.

	Full Sample N = 3084	White N = 2275	Black N = 443	Hispanic/Latino N = 364
Demographic Factors	N (%)	N (%)	N (%)	N (%)
Race/Ethnicity				
NH White	2,275 (73.82%)	----	----	----
NH Black	443 (14.37%)	----	----	----
Latino	364 (11.81%)	----	----	----
Education Level				
Less than HS	502 (16.28%)	228 (10.02%)	104 (23.48%)	169 (46.43%)
HS EQ	1,042 (33.79%)	796 (34.99%)	151 (34.09%)	95 (26.10%)
Some College	719 (23.31%)	528 (23.21%)	122 (27.54%)	68 (18.68%)
College or More	821 (26.62%)	723 (31.78%)	66 (14.90%)	32 (8.79%)
Relationship Status				
Married/Partnered	2,524 (81.84%)	1908 (83.87%)	313 (70.65%)	302 (82.97%)
Separated/Divorced/NevMarr	367 (11.90%)	219 (9.63%)	98 (22.12%)	49 (13.46%)
Widowed	193 (6.26%)	148 (6.51%)	32 (7.22%)	13 (3.57%)
	**Mean (SD)**	**Mean (SD)**	**Mean (SD)**	**Mean (SD)**
Age [Range: 40.8–99.1]				
Mean (SD)	66.76 (10.79)	68.08 (10.75)	62.98 (9.76)	63.15 (10.33)
Perceived Everyday Discrimination [Range: 1–4.8]				
Mean (SD)	1.56 (0.70)	1.53 (0.66)	1.71 (0.81)	1.61 (0.81)
**Health Factors**				
CES-D [Range: 0–8]^a^				
Mean (SD)	1.17 (1.81)	1.03 (1.72)	1.43 (1.79)	1.71 (2.22)
Number of Chronic Conditions [Range: 0–8] ^b^				
Mean (SD)	1.99 (1.47)	2.06 (1.47)	1.93 (1.50)	1.69 (1.43)
Body Mass Index [Range: 28.7–55.7]				
Mean (SD)	28.64 (5.07)	28.51 (4.99)	28.73 (5.21)	29.42 (5.34)
Activity of Daily Life Limitations [Range: 0–5] ^c^				
Mean (SD)	0.25 (0.72)	0.22 (0.68)	0.30 (0.82)	0.36 (0.83)
	**N (%)**	**N (%)**	**N (%)**	**N (%)**
Self-Rated Health				
Fair/Poor	761 (24.68%)	464 (20.40)	139 (31.38%)	157 (43.13%)
Good/Excellent	2,323 (75.32%)	1811 (79.60)	304 (68.62%)	207 (56.87%)
**Access Factors**				
Insurance Coverage				
Yes	2,691 (88.78%)	2094 (93.15%)	346 (80.28%)	250 (71.43%)
No	340 (11.22%)	154 (6.85%)	85 (19.72%)	100 (28.57%)
Primary Insurance Coverage				
Medicare/Medicaid/Public	1388 (53.28%)	1061 (53.18%)	188 (52.51%)	139 (55.38%)
Employer/Private	1071 (41.11%)	833 (41.75%)	141 (39.39%)	96 (38.25%)
TriCare/Va	146 (5.60%)	101 (5.06%)	29 (8.10%)	16 (6.37%)
Usual Place of Care				
Clinic/Health Center	438 (17.46%)	302 (16.06%)	69 (19.27%	67 (24.91%)
Doctor Office/HMO	1,828 (72.89%)	1474 (78.40%)	214 (59.78%)	139 (51.67%)
ER	76 (3.03%)	26(1.38%)	36 (10.06%)	14 (5.20%)
Outpatient	68 (2.71%)	26 (1.38%)	24 (6.70%)	18 (6.69%)
Other	36 (1.44%)	24 (1.28%)	4 (1.12%)	8 (2.97%)
no one place	62 (2.47%)	28 (1.49%)	11 (3.07%)	23 (8.55%)

In Table 1, The CES-D refers to the Center for Epidemiological Studies Depression (CES-D) Scale (a), while chronic conditions included hypertension, diabetes, cancer, chronic lung disease, heart conditions, stroke, psychiatric problems, and arthritis (b). Also, participants were asked if they had difficulty with any of five activities: bathing, dressing, eating, walking, and getting out of bed (c).

### The relationship between race/ethnicity and PCC

**Fig 1:**
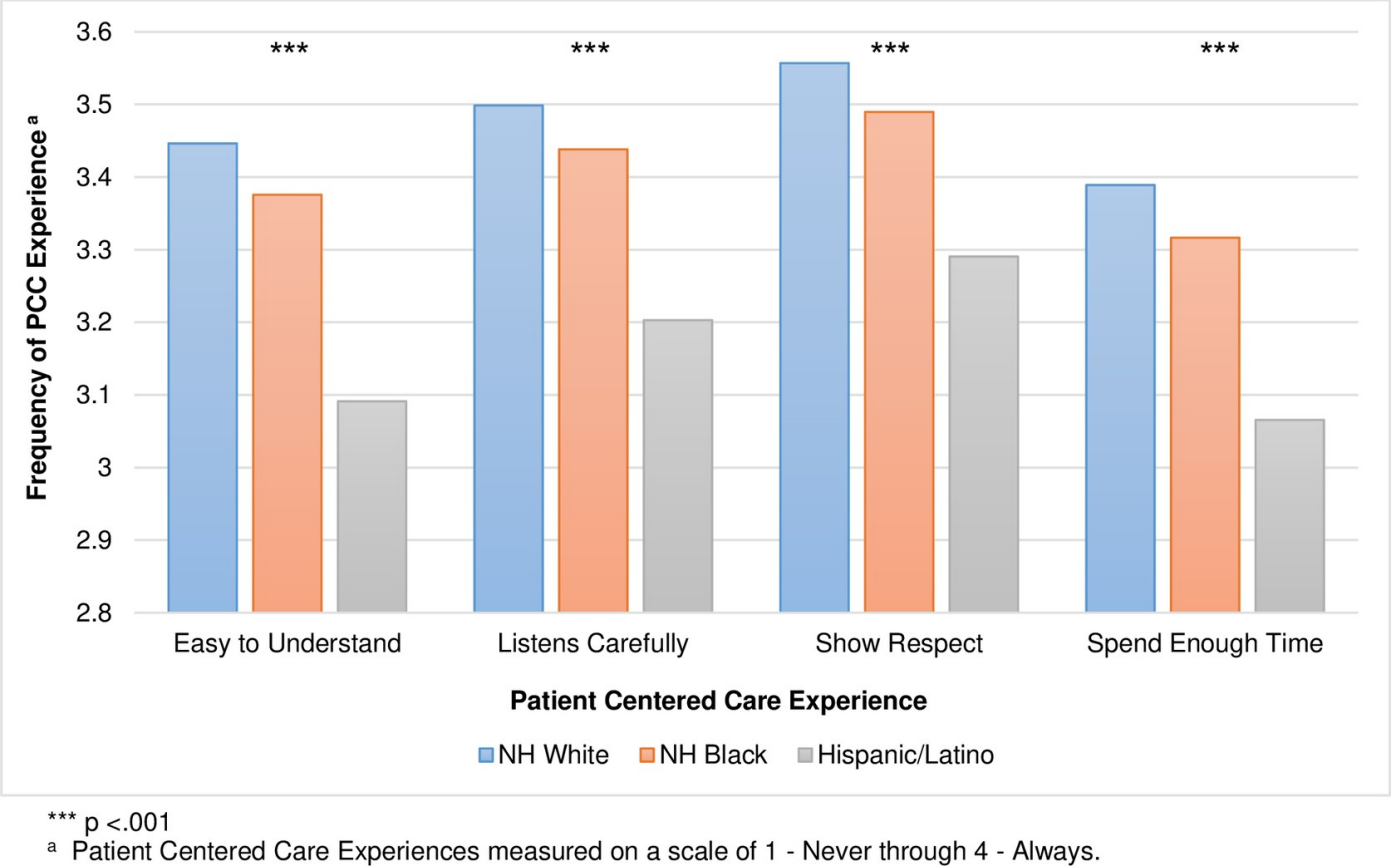
Reported Patient Centered Care (PCC) experiences by race/ethnicity.

A statistically significant MANOVA effect was obtained demonstrating one or more mean differences between Race/Ethnic groups (non-Hispanic White, Non-Hispanic Black, Hispanic/Latino) on reported PCC experiences, Wilks' lambda = .98, *F*(8, 5934) = 7.03, *p* < .001. The multivariate effect size was estimated at .1, which implies that 10% of the variance in the canonically derived dependent variable was accounted for by race/ethnicity. Post-hoc contrast analysis revealed that non-Hispanic White males’ mean was statistically significantly different from the average of Black and Latino males, Wilks' lambda = .98, *F* (4, 2967) = 9.58, *p* < .001. Subsequent contrast analysis also indicate regarding PCC experience, Black males had a statistically significantly different mean than Latino males in this study (see Fig 1).

### Predictors of PCC

Model 1 of the regression analysis consisted of race (non-Hispanic Black and Hispanic/Latino respondents, relative to White respondents), while Model 2 added sociodemographic, psychosocial, and physical health factors. In Model 3 health access factors were included. As expected, in Model 1, being Hispanic/Latino (relative to White respondents) was associated with, on average, a 0.3 point decrease on the PCC experience scale (B = -0.31; p < .001). Similarly, Black respondents scored roughly 0.06 points lower than White respondents (B = -0.06; p = .07), a marginally significant finding. These relationships were attenuated after accounting for biopsychosocial factors in Model 2, but remained significant for Hispanic/Latino respondents (B = -.14; p < .05). Psychosocial factors including discriminatory experiences (B = -0.16; p < .001), also negatively predicted PCC experiences. In model 3, healthcare access significantly predicted PCC experiences. Respondents lacking insurance coverage scored 0.64 points lower than those with insurance coverage (B = -0.64; p < .001). Also, those who held private insurance, relative to those with Medicare, scored lower in PCC (B = -0.14; p < .05). In model 3, facets of health were also predictive of PCC such that men who reported good or excellent self-perceived health also reported more PCC (B = 0.11, p < .05). Mental health was predictive in that each increase in reported symptoms on the CESD depression scale resulted in decreased PCC (B = -0.02, p < .05) While demographic factors such as education level, marital status, and age were not predictive of PCC in the final model, discriminatory experiences (B = -0.18, β = -0.21; p < .001) was the strongest predictor of PCC outcomes controlling for these and the remaining covariates, suggesting that discrimination may be central to differences in PCC experience.

### Predictors of PCC by race/ethnicity (Table 2)

In the final full model, analyses revealed that for Black men, discriminatory experiences predict lower reports of PCC (B = -0.44, P < .001). Chronic conditions positively predicted PCC for Black men such that reporting more chronic conditions predicted higher PCC (B = 0.10, p < .05). Insurance coverage also predicted PCC for Black men (B = -0.62, p = .05) such that Black men without insurance coverage reported less PCC than those with coverage. For Hispanic/Latino men, discriminatory experiences were not a significant predictor of PCC. The sole predictor of PCC for Hispanic/Latino men was having a place of usual care. For Hispanic/Latino men, having no place of usual care negatively predicted PCC experiences (B = -1.28, p < .01).

**Table 2:** Predictors of PCC (scaled) for men by race/ethnicity HRS, HCMS, PLBS 2010.

	NH White (N = 764)			NH Black (N = 117)			Hispanic/Latino (N = 69)		
	Coef.	Std. Err.	P>|t|	Coef.	Std. Err.	P>|t|	Coef.	Std. Err.	P>|t|
									
Perceived Everyday Discrimination	**-0.18**	**0.03**	**0.00**	**-0.44**	**0.09**	**0.00**	0.11	0.18	0.55
Education Level ^a^									
HS EQ	-0.09	0.08	0.28	0.07	0.16	0.64	0.31	0.32	0.33
Some College	-0.13	0.09	0.14	-0.10	0.18	0.56	0.23	0.32	0.47
College or More	-0.07	0.08	0.43	-0.07	0.20	0.72	-0.23	0.44	0.61
Relationship Status ^b^									
Separated/Divorced/NevMarr	0.14	0.08	0.10	0.15	0.14	0.29	-0.02	0.33	0.96
Widowed	0.07	0.09	0.43	0.24	0.23	0.28	-0.37	0.53	0.49
Age	0.00	0.00	0.23	0.00	0.01	0.67	0.02	0.02	0.15
CESD	**-0.03**	**0.01**	**0.05**	-0.03	0.04	0.38	-0.04	0.07	0.56
Chronic Conditions	0.00	0.02	0.96	**0.09**	**0.05**	**0.05**	0.04	0.10	0.73
Self-Rated Health ^c^									
Good/Excellent	**0.14**	**0.06**	**0.02**	-0.02	0.16	0.92	-0.08	0.28	0.78
BMI	0.01	0.00	0.16	-0.01	0.01	0.29	0.01	0.02	0.54
Activity of Daily Life Limitations	0.02	0.03	0.47	-0.05	0.08	0.56	-0.25	0.15	0.11
Insurance Coverage ^d^									
No	**-0.57**	**0.28**	**0.04**	**-0.63**	**0.32**	**0.05**	-0.77	0.43	0.08
Usual Place of Care ^e^									
Doctor Office/HMO	0.06	0.06	0.28	0.15	0.15	0.30	0.04	0.27	0.88
ER	-0.35	0.19	0.07	0.19	0.26	0.48	-0.43	1.05	0.69
Outpatient	0.10	0.17	0.58	-0.25	0.29	0.38	-0.58	0.52	0.27
Other	-0.18	0.18	0.32	-1.05	0.61	0.09	-1.58	0.92	0.09
no one place	**-0.55**	**0.16**	**0.00**	1.00	0.61	0.11	**-1.28**	**0.46**	**0.01**
Primary Insurance ^f^									
Medicaid	-0.04	0.25	0.88	-0.12	0.31	0.70	0.00	0.42	1.00
Employer provided	0.08	0.06	0.17	0.03	0.15	0.87	0.03	0.35	0.94
Private insurance	-0.11	0.07	0.15	-0.35	0.26	0.19	-0.32	0.51	0.53
Tri-Care/Champus	**0.39**	**0.18**	**0.03**	0.08	0.35	0.81	---	---	---
Other Public	-0.02	0.28	0.93	0.31	0.59	0.60	0.53	0.49	0.29
VA	**0.25**	**0.11**	**0.03**	0.46	0.42	0.29	0.85	0.60	0.16

In Table 2, the education level reference group is ‘ Less than high school’(a), while the relationship status reference group is ‘married/partnered’ (b). For the self-rated health, ‘fair/poor’ was the reference group (c) and for insurance coverage, ‘yes’ was the reference group (d). The reference group for place of care was ‘clinic or health center’(e).

## Discussion

As individuals age and their health care needs become potentially more complex, patient-provider communication serves as a critical bridge that supports individuals and families in advocating for themselves and navigating what can be a fragmented health care system. Here, we narrow our focus to Black and Latino men in the U.S., because research has identified them as two groups disproportionately burdened by chronic disease disparities, premature mortality, and less than optimal healthcare access and utilization. Therefore, it is particularly important to understand the degree to which these populations of men are receiving patient-centered communication during medical encounters and the multi-level factors that determine the patterning of such communication. The current study lends further support, richness, and depth to the limited existing knowledge base by disentangling how discrimination, health care access, and health status uniquely shape PCC for Black and Latino men in the U.S.

### The role of discrimination in PCC experiences for black men

For Black men in the current study, discriminatory experiences significantly predicted lower reports of PCC. A wealth of prior research has yielded important insights into the dangerous implications of experiencing racial discrimination for health outcomes among underrepresented minorities, and Blacks in particular [2, 13, 57]. When Black patients perceive that they are not being listened to, respected, and or given enough time to ask questions or interact with clinicians during medical visits, and that this treatment may be due to their race, studies show that they are more likely to mistrust providers, delay preventive care [16, 17], have poor glycemic control [58], and experience increased depressive symptomology [59]. Research clearly demonstrates that Black patients often attribute poor and ineffective clinical communication to discrimination; particularly when they perceive their health symptoms or perspectives are being overlooked or discredited during medical visits [40, 60]. This is a critical finding, when considered alongside the cumulative evidence base that both systemic racism [61] and perceived discrimination [62] are determinants of health, and in this study, they are determinants of health communication experiences for Black males. Perceived discrimination during health care interactions has similarly adverse impacts on Latino patients’ medical mistrust and health outcomes [37, 63], though this finding did not bear out in the current investigation and overall, the evidence base is more nascent than for Black patients in the U.S.

### Health access, health status, and PCC for black men

This study found that for Black men, reporting more chronic conditions predicted higher PCC scores, while Black men without insurance coverage reported less PCC than those with coverage. While other studies have found that multimorbid Black men may face increased barriers to patient-centered communication [24], it is also plausible that for the men in this study, having multiple health concerns provided more opportunities to discuss symptoms and feel listened to during medical visits. The effect of having a more complex health profile to discuss with physicians could also increase the perception that clinicians were spending enough time with these participants during medical visits. The finding that a lack of insurance coverage is associated with lower PCC scores is well-aligned with prior studies demonstrating that Black men with any type of health insurance coverage face fewer barriers than those who are uninsured in accessing PCC [24]. Prior studies dictate that health insurance is a reliable predictor of the quality of health care interactions [64]. One potential explanation is that those who are uninsured or under-insured may lack access to a usual source of care and out of necessity, be more likely to seek acute safety net care in emergency departments that are less patient-centered than primary care settings. Under this scenario, Rising and colleagues (2016) outline the challenges to patient-centered communication and care models in emergency departments,

“Finally, the substantial racial, ethnic, and socioeconomic diversity of patients and providers in the ED setting, and the need for providers to make cognitively efficient decisions to ensure safety of the entire population of ED patients seeking care at a given time, pose challenges to communication and delivery of high-quality, empathic emergency care.” [65, pg. 498].

Further, there is a psychosocial effect to having adequate health insurance coverage specifically among Black men. One study in particular reports that having health insurance increases Black men’s health self-efficacy, or their confidence in their ability to manage their own health [66]. These findings suggest that a lack of health insurance may not only disrupt the patient-provider communication dynamic, but may also cast a shadow over how Black men perceive their own capability for managing their health. It also seems important to acknowledge that since the enactment of the Affordable Care Act (ACA) in the U.S. in 2010, health insurance coverage has increased significantly for both Blacks and Latinos, though rates of uninsurance among both groups remains considerably higher than among Whites in the U.S [67]. Further, low income Black and Latino men residing in states without expanded Medicaid coverage under the ACA have likely not benefitted from reduced uninsurance rates or improved access to and stability of care.

### The intersection of health care access and PCC for Latino men

Thus far, extant research has not sufficiently investigated the factors that underlie suboptimal health communication experiences between Latino men and their health providers. We partially address this gap in knowledge with the final analytic model of this study, showing that the sole predictor of patient-centered communication scores for Latino men is having a place of usual care. A usual place of care denotes a relationship with a non-emergency health provider (e.g. primary care) that can often oversee continuous and comprehensive routine, preventive, and chronic disease health care. Latino men in the U.S. without access to a usual source of care are lacking in the type of patient-provider relationships that support ongoing patient engagement in decision-making, fosters more positive patient experiences, and reduces disparities in screening for common chronic conditions and cancer at the point of care, relative to other Latinos and Whites without a usual source of care [56].

There are numerous potential interpretations of this finding, many beyond the scope of this paper, but certainly important to note. First, having a usual place of care is often predicated on having adequate health insurance and up to 34% of Latino Americans lack health insurance [1]. It is also established that having a usual source of care and related healthcare utilization may be structured by socioeconomic status, and lower socioeconomic status is a well-known proxy for certain social and environmental exposures that can negatively impact health among Latino men [68]. Among some Latino men in the U.S., limited English proficiency may also pose a challenge to both navigating the health care system (e.g. making appointments) and engaging in PCC. While it was not a significant predictor of PCC in the current study, the added burden of institutional and interpersonal discrimination is a psychosocial stressor that has been demonstrated to diminish Latino men’s engagement with healthcare settings [1, 69]. Taken together, these factors paint a complicated and interconnected image of how Latino men encounter and engage with health care systems and in this context, health communication with providers. Renewed research attention needs to be given how patient-centered care and communication are enacted upon Latino men, including both gender- and ethnic-specific experiences (e.g. male ethnic sub-group differences) and the role that geography, migration, acculturation and other factors play in patterning health care interactions and engagement.

## Conclusion and limitations

For both Black and Latino men in this study, the impact of social determinants on men’s experience of patient-centered communication cannot be overstated. It is evident from these results that some of the very factors or conditions that promote or inhibit health risks and a wide range of outcomes among men (i.e. health access, health insurance, and perceived discrimination), also extend into the patient-provider encounter for Black and Latino men in the U.S. When these factors are combined with socio-cultural constructions of gender and masculinity that constrain men’s health help-seeking [70–72], and inhibit men’s ability to communicate effectively with providers [73], Black and Latino men are left at a significant disadvantage during medical interactions. This compounded dynamic further perpetuates health inequities. Based upon the above discussion, this study applies a patient-centered communication lens to elucidating the underlying relationships between multilevel and interconnected factors such as health status and coverage, perceived discrimination, and health care access for Black and Latino men. This study also provided missing detail on the specific link between having a usual source of care and disparities in patient-centered communication for heterogenous Latino men in the U.S., and added context linking perceived racial discrimination to lower scores of patient-centered communication for Black men. This study accentuates the need for additional research on Latino men’s experiences with PCC in particular, given the notable gaps in extant literature. Ultimately, these findings can be used to inform the development of needed additional research, clinical tools, interventions and health care processes targeting improvements in the experience and outcomes related to PCC for minority males.

These findings should be interpreted in light of a few key limitations. First, this research used a cross-sectional design. Future iterations of this work could employ a longitudinal design to better establish the causal directions in the relationships between race, socio-demographic factors, and PCC. A cross-sectional analysis is also unable to provide necessary context on Black and Latino men’s health engagement and relationship to PCC in the context of medical encounters over time. Also, population-based samples such as the Health and Retirement Study often sample smaller proportions of ethnic minorities. This study had a relatively small sample of Hispanic/Latino respondents which may have impacted the study’s power for detecting PCC trends among Hispanic/Latino respondents. Further, the authors did not account for any potential interaction in the analysis between age and activities of daily living, and while those variables were not significant to the experience of PCC in this sample, future researchers may want to explore whether minority men who do have more functional difficulties experience increased barriers to PCC. In addition, relying on a secondary analysis of existing data that potentially under samples minority men across the U.S. may belie regional differences in the diversity of social and health care conditions for minority men in vastly different U.S. geographies. The current study was also not able to account for the potential impact of immigration, English language use and proficiency during medical encounters, acculturation of Latino males, or whether factors such as family support impact the receipt of PCC during medical visits for both Black and Latino males. While the current study did include a discussion of the potential intersection of gender norms and socialization on health care utilization and experiences, the study’s analysis could not account for these factors as well. These limitations notwithstanding, our findings suggest that Black and Latino males need better support in effectively connecting with and communicating their questions and concerns during medical visits as they age, in addition to culturally tailored tools for self-advocacy in a constantly-shifting and fragmented health care system.
